# User Acceptance of a Virtual Reality Beer Pong Intervention in Postoperative Rehabilitation: Exploratory Mixed Methods Study

**DOI:** 10.2196/82704

**Published:** 2026-08-06

**Authors:** Tim Schneider, Merle Schlender, Verena Uslar, Sirko Pelzl, Daniela Salzmann, Nomi Anna Kreß, Navid Tabriz, Dirk Weyhe

**Affiliations:** 1 Department for Human Medicine, University Hospital for Visceral Surgery, PIUS-Hospital, Faculty VI, University of Oldenburg, Ammerländer Heerstraße 114-118, Oldenburg, 26129, Germany, 49 4412292922; 2 apoQlar medical GmbH, Hamburg, Germany

**Keywords:** immersive therapy, human-computer interaction, patient engagement, digital health, gamification, technology acceptance

## Abstract

**Background:**

Postoperative patients often face pain, reduced mobility, and motivational barriers, while health care systems increasingly struggle with shortages of physiotherapy staff, making the evaluation of digital rehabilitation tools highly relevant. In this context, virtual reality (VR)–based rehabilitation has already shown promise in neurological and musculoskeletal settings, but evidence regarding its acceptance and usability in postoperative inpatient rehabilitation, particularly in surgical settings with potentially older, multimorbid patients, as well as the general acceptance and usability of VR-supported physiotherapy, is still insufficiently investigated.

**Objective:**

This exploratory mixed methods study investigated the usability and acceptance of a VR-based rehabilitation application among postoperative patients and physiotherapists in a clinical setting. Secondary objectives were to gather initial data on potential differences in usability across age and gender groups and to explore patients’ and physiotherapists’ attitudes toward VR as a supplement or replacement for conventional physiotherapy.

**Methods:**

This cross-sectional exploratory mixed methods study with prospective participant recruitment and data collection included 35 postoperative inpatients (17 female participants and 18 male participants; age groups: <40, 40‐60, and >60 years) and 7 physiotherapists (4 female participants and 3 male participants) recruited from visceral surgery and orthopedic departments. Participants used a VR-based rehabilitation demonstrator (“Beer Pong,” apoQlar GmbH) delivered through a head-mounted display (Meta Quest 3) for a standardized 5-minute session involving upper-limb movements and squatting exercises. The System Usability Scale (SUS) and a self-developed questionnaire were used to assess the usability of the whole system and general attitudes toward VR-supported physiotherapy. Usability ratings were analyzed descriptively and using a 2-way ANOVA to test for effects of age group and gender. Descriptive statistics were used to summarize responses on motivation, distraction, supplementation, and preferences regarding VR use.

**Results:**

The overall mean SUS score was 73.21 (SD 13.74, 95% CI 68.49-77.93). No significant differences in usability ratings were found between age groups or genders. Most patients (29/35, 83%) agreed that the VR scenario increases motivation, and 92% (32/35) viewed VR exercises as a useful supplement to conventional physiotherapy. Physiotherapists reported an average SUS score of 74.64 (SD 10.04, 95% CI 65.35-83.93) and expressed positive attitudes toward VR implementation, as assessed using a self-developed questionnaire. However, only 20% (7/35) of patients believed VR could replace traditional therapy entirely.

**Conclusions:**

The results of this pilot study seem to indicate high acceptance of VR-based rehabilitation applications by patients and physiotherapists in the clinical context of a postoperative setting. As one of the first studies to evaluate VR usability specifically in early postoperative inpatient rehabilitation, this work extends existing evidence beyond previously studied rehabilitation domains. Taken together with the possible potential to increase patients’ motivation while relieving work strain on physiotherapists, these findings support further investigation through larger controlled trials to assess long-term usability, clinical efficacy, and integration into routine postoperative clinical care, even for older people.

## Introduction

### Virtual Reality in Postoperative Rehabilitation

Postoperative rehabilitation is of the greatest importance for restoring function and relieving pain after surgery. Successful rehabilitation is instrumental in improving recovery and quality of life in patients [1]. Systematic physical training tailored to an individual patient’s postoperative condition, functional capacity, and surgical limitations is central to these efforts. Weyhe et al [2], for example, demonstrated in a randomized controlled trial that supervised physiotherapy or home-based exercise initiated early after the resection of pancreatic cancer is both feasible and beneficial in the postoperative course.

In recent years, virtual reality (VR) has emerged as a promising technology to enhance physical rehabilitation [3]. VR exposes people to computer-generated environments that provide multimodal sensory feedback in an attempt to increase motivation, engagement, and adherence to exercise routines [4]. Moreover, VR is increasingly regarded as a potential solution to support the delivery of high-quality physiotherapeutic care in light of the growing shortage of skilled rehabilitation professionals [5].

Evidence for the applicability of VR-assisted rehabilitation is increasing. Evaluating applicability in this context often includes measures of improvement in patients’ performance and motivation, as well as the usability of the developed system. In fact, a recent systematic review by Brepohl and Leite [6] revealed several advantages with regard to those outcomes, including, for instance, improved balance, gait, postural control, and pain, as well as enhanced motivation and training performance [7-9]. However, several obstacles need to be overcome: costly technology, restricted availability of suitable rehabilitation games, technical issues like sensor inaccuracy, lack of standardization, and lower familiarity among physiotherapists may hinder extensive use [6,10-13].

To date, the majority of VR applications in rehabilitation have been introduced in neurology (54%), followed by orthopedics and trauma treatment (14%) [6]. VR-based treatments are generally intended to augment, rather than substitute for, traditional physiotherapy and are best customized to address individual patient requirements [6,14].

In patients who have experienced neurological impairments, evidence suggests that VR can support improvements in specific motor functions of the upper limb. For example, a systematic review by Darekar et al [15] found that, in children with cerebral palsy, VR interventions were compared with conventional physiotherapy—defined as therapist-led, non-VR upper-limb training, including activities such as reaching, grasping, and manipulation exercises measured with tasks like the Quality of Upper Extremity Skills Test and grip strength assessments—and were associated with positive changes in upper limb motor outcomes, including range of motion, coordination, and manual function across several randomized trials, although overall evidence certainty remains low and results varied among studies. Similarly, findings by Darekar et al [15] indicated that VR-based rehabilitation can lead to improvements in balance, mobility, walking speed, and the ability to navigate complex environments following a stroke. A recent systematic review by Wang et al [16] also identified enhancements in gait and balance in individuals with spinal cord injuries as a result of VR interventions. However, the authors emphasize that the current body of evidence remains limited, highlighting the need for further research in this area. In the context of Parkinson disease, Yu et al [17] reported a significant positive effect of VR-based therapy on balance. Moreover, a study by Lin et al [18] found that active video game–assisted therapy led to notable reductions in pain as well as improvements in mobility and functional ability in patients with musculoskeletal disorders.

With regard to motivation, it is known that patients perceive rehabilitation using video games as more enjoyable and less taxing compared to traditional programs [18,19]. This holds true even for an older patient population [20]. However, it is not known if this applies to user acceptance of VR applications as well, particularly in a surgical rehabilitation setting with early postoperative patients and clinical end users such as physiotherapists. In this context, a surgical rehabilitation setting refers to the early postoperative phase of inpatient hospital care, where physiotherapy is delivered under medical supervision and might possibly be constrained by surgical precautions, pain, fatigue, and limited mobility.

In addition to patient improvement and a decrease in motivation, an important aspect in the evaluation of VR-based rehabilitation applications is the usability of the application itself. This might encompass the hardware used for the application, as well as the application itself, or the combination of both. The System Usability Scale (SUS) has emerged as a simple tool for measuring the usability of VR applications [20], which has been validated in a number of contexts [21,22]. In recent research, the SUS was, for instance, used to evaluate a VR mirrored hand system for stroke patients or a VR application designed to work with an omnidirectional treadmill [23,24]. However, usability is mostly evaluated from the patients’ point of view, whereas physiotherapists are seldom involved in the development of a VR application from the start. This might be at least one of the reasons why there is no widespread usage of VR applications [25]. Recent work has emphasized the importance of clinician involvement in the development of VR rehabilitation tasks. For example, Danso et al [26] reported on the codevelopment of a VR task with physiotherapists for poststroke visuospatial neglect, highlighting how therapist-driven design can enhance task relevance, usability, and user experience.

To summarize, especially in light of the decreasing availability of physiotherapists for postoperative mobilization, digital support systems based on VR applications seem to have the potential to increase rehabilitation success, as well as patient motivation. Therefore, they may play an increasing role in guiding rehabilitation exercises. However, currently, little is known about the applicability of and the general attitude toward VR rehabilitation applications in an early postoperative clinical setting, especially from the viewpoint of a mostly older multimorbid surgical patient population, as well as clinical physiotherapists. Therefore, before starting a larger intervention study at the University Hospital for Visceral Surgery, PIUS-Hospital Oldenburg, we aimed to evaluate whether a typical surgical patient population in a university hospital would generally be willing to receive support during postoperative inpatient physiotherapy through a VR demonstrator and whether clinically working physiotherapists would be willing to offer such applications to their patients and recognize a potential added value in them. To help patients and physiotherapists better understand the concept, we tested the demonstrator “Beer Pong VR” by apoQlar GmbH.

### Study Aim and Outcome Measures

VR-based rehabilitation can be understood as a complex intervention, as it combines technological components, user interaction, and clinical context. This study was therefore designed as an exploratory mixed methods test focusing on usability and acceptance as early-stage outcomes. In line with the early development and feasibility phases of complex intervention research, the aim was not to assess clinical effectiveness but to evaluate whether the intervention is usable, acceptable, and suitable for further investigation in postoperative rehabilitation settings in order to ascertain the feasibility of conducting a future full-scale clinical trial, which could then incorporate measurements of clinical benefit as well.

With this in mind, the primary outcome of this exploratory pilot study was the perceived usability of an exemplary integrated VR rehabilitation system as a whole, measured using the SUS, in patients depending on age and in physiotherapists.

Secondary descriptively analyzed outcomes were as follows: (1) general patients’ and physiotherapists’ attitudes toward VR-based exercises compared with video-based exercise formats, assessed using a self-designed questionnaire; (2) perceived reasons for the acceptance or rejection of VR-supported training; and (3) a comparison of pain levels before and during the exercise.

## Methods

### Ethical Considerations

The study was conducted as human participants’ research in accordance with national regulations, the principles of the Declaration of Helsinki (1975), and the guidelines of Good Clinical Practice (ICH-GCP). Prior to study initiation, ethical approval was obtained from the responsible Medical Ethics Committee (reference number 2024‐164). In addition, approval was granted by the Data Protection and Information Security Management Unit at the University of Oldenburg and the staff council at PIUS-Hospital. Written informed consent was obtained from all participants prior to inclusion. Patients and physiotherapists were informed verbally and in writing about the study objectives, procedures, potential risks (eg, dizziness during VR use), data handling, and their right to withdraw at any time without consequences for their treatment or employment. No waivers of consent were applied. Participation was voluntary, and no compensation was provided. Recruitment was carried out by medical staff from the Department of Visceral Surgery at the University of Oldenburg, located at PIUS-Hospital in Oldenburg, through personal communication. To ensure privacy and confidentiality, all collected data were pseudonymized immediately after data acquisition. No directly identifiable personal information was stored together with study data. The pseudonymized dataset was stored on a password-protected computer system, with access restricted to authorized members of the research team only. Data processing complied with applicable data protection regulations. No images, videos, or supplementary materials included in the manuscript allow for the identification of individual participants. The figures presented depict anonymized data visualizations or screenshots of the virtual environment without personal identifiers. Therefore, no additional consent for the publication of identifiable images was required.

### Participants

Data were collected between November and December 2024. A total of 35 postoperative patients hospitalized during this period (Table 1) and 7 physiotherapists (4 female participants and 3 male participants) participated in the study. Patients were categorized into 3 age groups for analysis: less than 40 years, 40 to 60 years, and 60 years and older.

**Table 1.** Patient characteristics of postoperative participants enrolled in a pilot study evaluating a virtual reality (VR)–based exergaming intervention (Beer Pong) in a clinical rehabilitation setting, including gender, physical activity, diagnosis, treatment type, and VR experience.

Patient characteristics	Less than 40 years (n=10)	40‐60 years (n=12)	60 years and older (n=13)	Patients (N=35)
Gender				
Female	3	8	6	17
Male	7	4	7	18
Physical activity				
None	3	4	3	10
Various^a^	7	8	10	25
Category of condition (topographical)				
Abdominal^b^	5	6	7	18
Upper extremity^c^	1	1	3	5
Lower extremity^d^	3	4	2	9
Collum^e^	0	0	1	1
Regio gluteales^f^	1	1	0	2
Type of therapy (invasiveness)				
Minimally invasive	6	6	4	16
Open	3	5	8	16
Other^g^	1	1	1	3
Previous experience with VR^h^				
<5 min	1	0	0	1
>20 min	6	1	1	8

a Fitness, cycling, hiking, Hot Iron, Qi Gong, tennis, golf, swimming, yoga, rowing, squash, football, and Pilates.

b Appendicitis, pancreatic carcinoma, cholecystolithiasis, rectal carcinoma, malignant perforation of internal organs, gastric carcinoma, metastatic breast carcinoma, adrenal adenoma, and unclear abdominal pain.

c Shoulder joint arthrosis, biceps tendon rupture, and finger fracture.

d Femoral head necrosis, dislocated knee prosthesis, knee joint arthrosis, coxarthrosis, and ankle joint fracture.

e Thyroid nodules.

f Pilonidal sinus or abscess.

g Abscess incision.

h The remaining participants were categorized as having no previous experience with VR.

Among the physiotherapists, 3 reported prior experience with VR (2 for <5 min and 1 for >20 min). Recruitment proved challenging, primarily due to patients reporting a subjectively poor general condition, significant disease-related distress, or acute pain at the time of recruitment. Participants were recruited through consecutive sampling during routine postoperative inpatient care. Eligible patients were identified by clinical staff from the departments of visceral surgery and orthopedics and were approached in person during their hospital stay.

Patients were eligible for inclusion if they met the following criteria:

- Age 18 years and older
- Current postoperative inpatient care
- Ongoing physiotherapeutic treatment
- Ability to perform physical exercises
- Medical clearance for mobilization by a physiotherapist
- Provision of written informed consent

Patients were excluded if any of the following applied:

- Preexisting neurological conditions (eg, stroke, multiple sclerosis, Parkinson disease, amyotrophic lateral sclerosis, or epilepsy)
- Muscular disorders affecting physiotherapy participation (eg, muscular dystrophy or myasthenia gravis)
- Inability to tolerate VR headsets (eg, dizziness or nausea)
- Severe postoperative complications (eg, sepsis, significant bleeding requiring reoperation, bowel obstruction, or respiratory failure requiring intensive care)
- Insufficient German language proficiency
- Inability or unwillingness to provide written informed consent

Physiotherapists were eligible for inclusion if they fulfilled the following criteria:

- Provision of written informed consent

Physiotherapists were excluded if they met the following criteria:

- Unable to tolerate VR
- Insufficient German language proficiency
- Unwilling or unable to provide written informed consent

### Procedure

**Figure 1.**
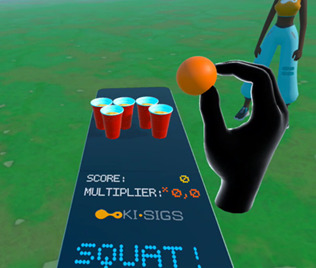
Virtual reality (VR)–based exergaming demonstrator (“Beer Pong”) used for postoperative physiotherapeutic exercises. Illustration of the immersive VR exergame used in this pilot study. To interact with the virtual ball, users are required to perform a squat, thereby combining lower-body activation with goal-directed upper-body movements and coordination to support postoperative physiotherapeutic rehabilitation.

Participants were introduced to the VR system, which consisted of the Meta Quest 3 headset and the “Beer Pong” demonstrator developed by apoQlar GmbH (Figure 1). This introduction included a structured familiarization phase during which the experimenter explained the VR headset, the application interface, and the task sequence in detail. Verbal instructions were provided in an explanatory and supportive manner but were not strictly standardized. Core task elements, including performing a squat before each throw, grasping the virtual ball, and throwing it into the cup, were explained consistently, while additional guidance was adapted to individual participants’ needs and questions. Participants then completed 3 practice throws to familiarize themselves with the interaction mechanics. During this phase, the VR display was mirrored to a laptop to identify and resolve potential technical or usability issues. The participants wore the VR headset and engaged with the app for 5 minutes. In the game, users were required to grasp a virtual ball and throw it into cups. Before each throw, the application prompted them to perform a squat, with movement execution monitored and, if necessary, corrected by physiotherapists to address improper posture or unsafe movement patterns. Users interacted with the system using hand-based gesture control to grasp and throw the virtual ball. Motion tracking was performed with the inbuilt sensors of the Meta Quest 3 headset.

Immediately after the session, participants completed the SUS to assess perceived usability. In addition to the SUS, a custom-designed questionnaire was administered to assess acceptance and motivation among both patients and physiotherapists, using a 5-point Likert scale ranging from “strongly disagree” to “strongly agree.”

### System Usability Scale

The SUS is a validated method for measuring the subjective perceived learnability and user satisfaction of new technological applications. The SUS consists of 10 items, each rated on a 5-point Likert scale ranging from 1 (strongly disagree) to 5 (strongly agree). For odd-numbered items, the score is adjusted by subtracting 1, whereas for even-numbered items, the score is adjusted by subtracting the item rating from 5. This scoring procedure results in a value between 0 and 4 for each item. The scores of all items are then summed and multiplied by 2.5 to yield a total SUS score ranging from 0 to 100. This score is referred to as the system usability score [20]. It is important to note that the average SUS score across systems is 68, while scores of 80 or above fall within the top 10 percentile. Based on these thresholds, scores can be categorized as follows: 0‐51.6=poor, 51.7‐62.6=OK, 62.7‐72.5=good, 72.6‐78.8=excellent, and 78.9‐100=the best imaginable [21]. The full SUS questionnaire is provided in Multimedia Appendix 1. In this study, the SUS was used to assess the perceived usability of the integrated VR rehabilitation system as a whole, rather than a single isolated component.

Specifically, participants were instructed to evaluate their overall experience with the combined system, including the Meta Quest 3, the Beer Pong demonstrator, and the basic interactions required to perform the exercises within a supervised clinical setting.

Usability ratings, therefore, reflect the participants’ perception of the combined hardware-software system as experienced during the standardized intervention session and not the usability of the software application or hardware device in isolation.

### Acceptance and Attitudinal Questionnaire

In addition to the SUS, a self-developed questionnaire was used to assess patient and physiotherapist attitudes toward VR-supported physiotherapeutic exercises. The questionnaire addressed motivation, perceived distraction, preferences compared with conventional exercise formats, the perceived role of VR as a supplement or replacement for standard physiotherapy, and willingness to use VR-based exercises in a home setting (Multimedia Appendix 1).

The questionnaire was designed as an exploratory instrument to capture context-specific user perceptions relevant to postoperative rehabilitation. Given the exploratory nature of the study and the limited sample size, the items were intentionally descriptive, focusing on clinically relevant aspects of user experience rather than formal theoretical constructs. Accordingly, the results derived from this questionnaire are intended to provide an initial descriptive overview of user acceptance and should be interpreted as exploratory and hypothesis-generating.

### Statistics

#### Quantitative Analyses

The quantitative SUS data were analyzed descriptively and using ANOVA to examine the effects of age and gender. To explore within-subject differences in pain ratings before versus during the VR exercise session, a Wilcoxon signed-rank test was conducted. This nonparametric test was selected due to the ordinal nature of the pain scale and the paired structure of the data. Effect size was calculated as *r* = *Z*/√n. Given the pilot nature of the study and the limited sample size, these analyses were intended to be descriptive and hypothesis-generating rather than confirmatory; therefore, no formal power analysis was conducted. Data analysis was conducted using SPSS (version 28.0.1.0; IBM Corp), MATLAB (version R2022a; The MathWorks Inc), and Microsoft Excel (version 2408, Microsoft Corporation, 2024).

#### Qualitative Analyses

Open-ended responses were analyzed using a descriptive qualitative approach. Responses were reviewed, grouped by recurring themes (eg, motivation, perceived distraction, and usability-related comments), and summarized narratively. No formal qualitative coding framework or theory-driven qualitative methodology was applied for the 2 questions posed to patients (“Do you have any suggestions or requests?” and “Further comments?”) or physiotherapists (the same questions and in addition: “In your opinion, what could the integration of VR-supported physiotherapy into everyday life look like?”). However, we roughly followed the thematic analysis framework [27]. Since our literature review prior to the study revealed no standardized or validated questionnaires that assess patients’ or physiotherapists’ attitudes toward the use of VR in physiotherapy, we developed our own questionnaire. The questions were initially developed by the study team with particular attention to clarity, relevance, and comprehensibility for the target groups. These questions were then presented to potential patients and the head of the physiotherapy department, who provided feedback and, if necessary, adjusted to improve clarity and ensure all relevant aspects were covered. The questionnaire was developed for exploratory purposes in this pilot study and did not undergo formal psychometric validation. Therefore, findings derived from these items should be interpreted cautiously and regarded as descriptive.

## Results

**Figure 2.**
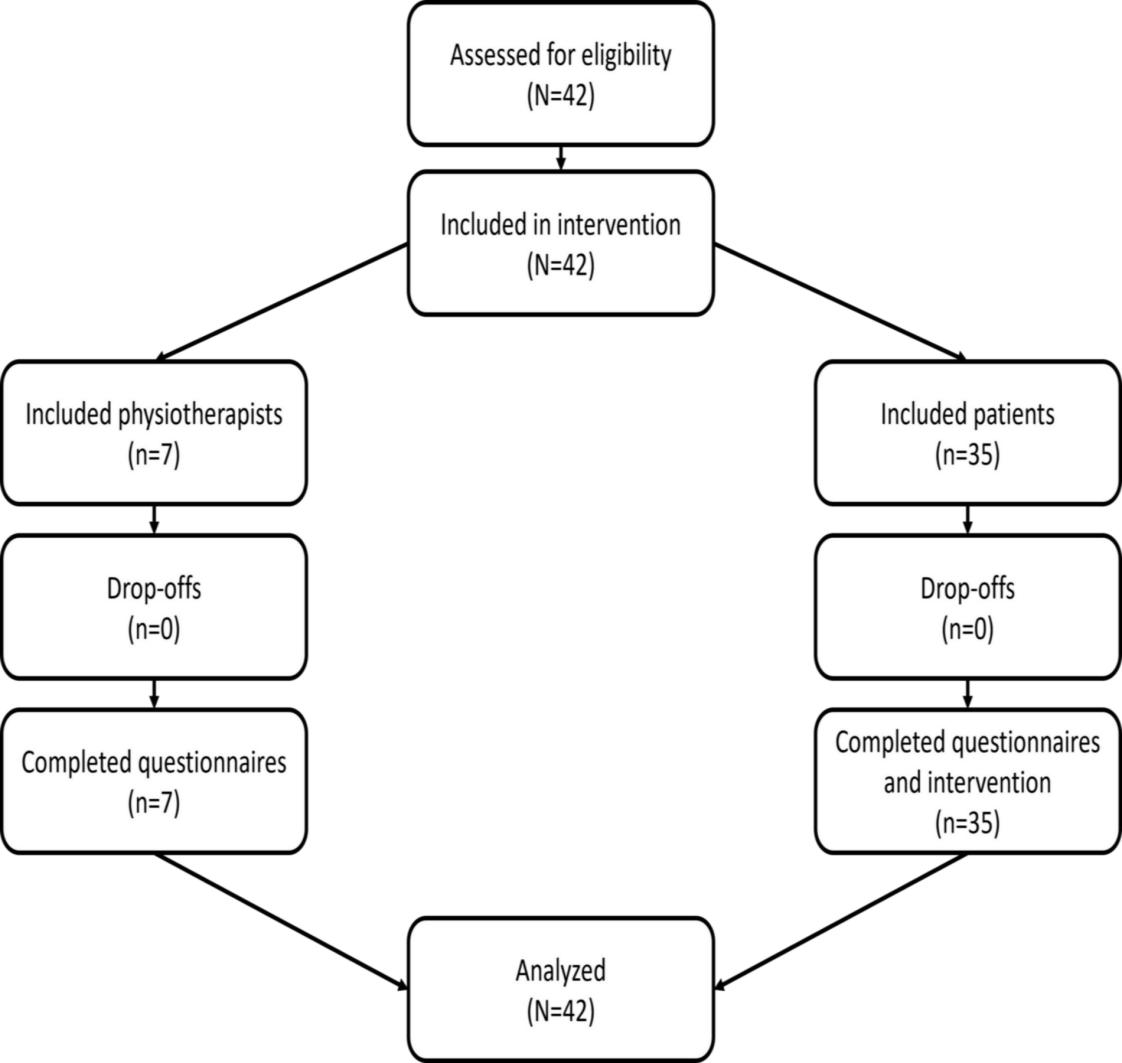
Flowchart of participant progression through the study, including eligibility assessment, allocation to intervention, completion status, and final analyzed sample for patients and physiotherapists.

**Figure 3.**
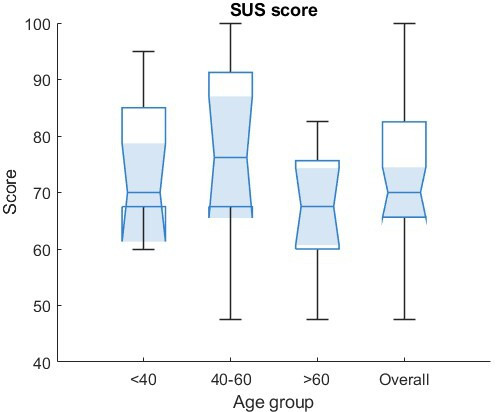
System Usability Scale (SUS) scores of the virtual reality–based exergaming application stratified by age group. Boxplots show SUS scores for postoperative patients grouped by age (<40, 40‐60, >60 years) and for the overall study sample. Boxes represent the IQR, horizontal lines indicate median values, and whiskers depict the range of observed scores. Higher scores indicate greater perceived usability of the virtual reality application.

The participant flowchart is presented in Figure 2. The SUS was used to assess the perceived usability of the VR application across all participants. The overall mean SUS score was 73.21 (SD 13.74, 95% CI 68.49-77.93). When stratified by age group, participants under 40 years reported a mean SUS score of 75.75 (SD 12.36, 95% CI 66.91-84.59), those aged 40 to 60 years had a mean score of 77.71 (SD 15.54, 95% CI 67.84-87.58), and participants over 60 years reported a mean score of 67.12 (SD 11.54, 95% CI 60.14-74.09). A visual comparison of SUS scores across age groups is provided in Figure 3. In addition, exploratory inferential analyses were conducted using a 2-way analysis of variance to examine potential effects of age group and gender. Homogeneity of variances was not violated (Levene test, *P*=.84). The exploratory analyses did not indicate statistically significant differences in usability ratings between age groups or genders (*F*_1,29_=0.30, *P*=.59, partial *η*²=0.010). Similarly, there was no statistically significant effect of age group on patients’ SUS scores (*F*_2,29_=2.72, *P*=.08, partial *η*²=0.158).

**Figure 4.**
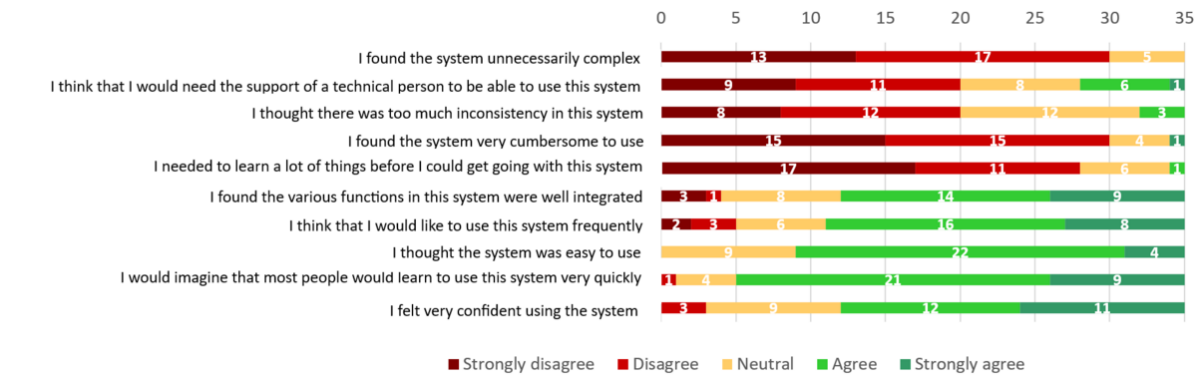
Distribution of patient responses to individual System Usability Scale (SUS) items for the virtual reality–based exergaming application. A stacked bar chart shows responses from postoperative patients (N=35) across all 10 SUS items. The upper 5 items are negatively worded, and the lower 5 items are positively worded; responses are displayed on a 5-point Likert scale ranging from “strongly disagree” to “strongly agree.” Higher agreement with positively worded items and lower agreement with negatively worded items generally indicate higher perceived usability. The figure illustrates an overall tendency toward favorable usability perceptions across most items.

An overview of the response distribution for each of the 10 items of the SUS is presented in Figure 4. The bar chart differentiates between negatively worded items (items 1-5) and positively worded items (items 6-10). The figure illustrates the number of participants selecting each response option on the 5-point Likert scale, ranging from *strongly disagree* to *neutral* to *strongly agree*, thereby providing insight into specific aspects of perceived usability.

Thirty out of 35 (86%) patients disagreed with the statement that the system is unnecessarily complex. Twenty-three (66%) patients agreed that they felt confident using the system, while only 3 (9%) disagreed. Additionally, 24 (69%) patients expressed a willingness to use the system more frequently, with 6 (17%) remaining neutral.

**Figure 5.**
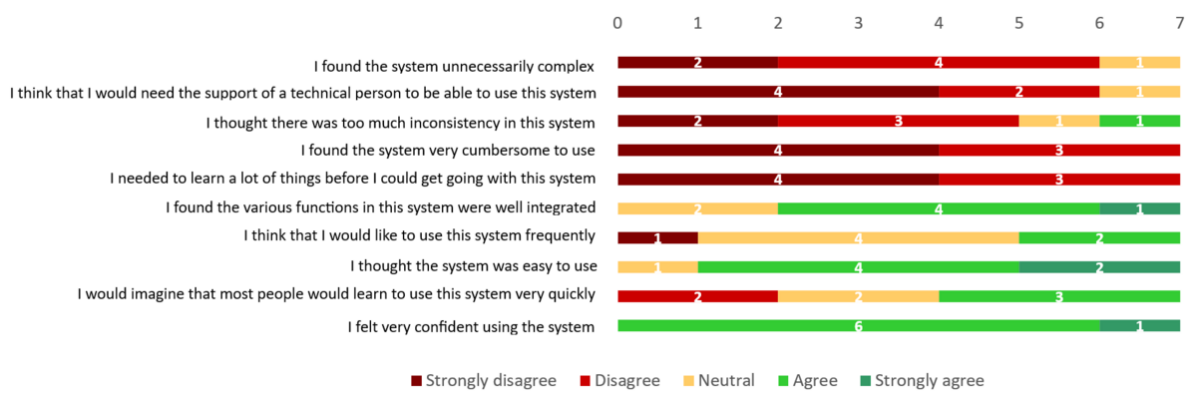
Distribution of physiotherapist responses to individual System Usability Scale (SUS) items for the virtual reality–based exergaming application. A stacked bar chart shows responses from physiotherapists (n=7) across all 10 SUS items. The upper 5 items are negatively worded, and the lower 5 items are positively worded; responses are displayed on a 5-point Likert scale ranging from “strongly disagree” to “strongly agree.” Higher agreement with positively worded items and lower agreement with negatively worded items generally indicate higher perceived usability. The figure illustrates an overall tendency toward favorable usability perceptions across most items.

The SUS scores obtained from the participating physiotherapists yielded a mean score of 74.64 (SD 10.04, 95% CI 65.35-83.93). The evaluation of the SUS responses from the participating physiotherapists is illustrated in Figure 5. Similar to the patient data, the first 5 items represent negatively worded statements, while the lower 5 items are positively phrased. The figure provides insight into how consistently the physiotherapists perceived the usability of the VR system across individual aspects, ranging from “strongly disagree” to “strongly agree.” Six out of 7 (86%) disagreed with the statement that the system is unnecessarily complex. All 7 physiotherapists agreed that they felt confident using the system. Regarding the intention to use the system more frequently, 1 (14%) physiotherapist strongly disagreed, 2 (29%) physiotherapists agreed, while 4 (57%) remained neutral, neither agreeing nor disagreeing.

Twenty-nine out of 35 (83%) patients agreed or strongly agreed that diverse VR scenarios would enhance their motivation to practice. Thirty-three (94%) participants found VR exercises to be a welcome distraction from the hospital routine. Additionally, 32 (92%) patients could envision using VR-based exercises as a supplement to conventional physiotherapy. However, only 7 (20%) patients agreed that VR could replace traditional physiotherapy, with a considerable portion remaining neutral or disagreeing. Furthermore, 27 (77%) patients expressed willingness to perform VR-supported physiotherapeutic exercises at home. These results, obtained from a self-developed questionnaire, are summarized in Table 2.

**Table 2.** Patient-reported attitudes toward virtual reality (VR)–supported physiotherapeutic exercises in a postoperative pilot study (N=35), evaluating acceptance of a VR-based exergaming intervention for physiotherapeutic rehabilitation^a^.

Question	Strongly disagree, n (%)	Disagree, n (%)	Neutral, n (%)	Agree, n (%)	Strongly agree, n (%)
Different VR scenarios for practicing would increase my motivation	1 (2.9)	1 (2.9)	4 (11.4)	19 (54.3)	10 (28.6)
Performing exercises using VR provides a welcome distraction from everyday hospital routines	—^b^	1 (2.9)	1 (0.03)	13 (37.1)	20 (57.1)
I can imagine using VR-based exercises as a supplement to conventional physiotherapy	—	1 (0.03)	2 (5.7)	15 (42.9)	17 (48.6)
I can imagine using VR-based exercises as a replacement for conventional physiotherapy	4 (11.4)	9 (25.7)	15 (42.9)	6 (17.1)	1 (2.9)
I prefer VR over traditional alternatives, such as watching exercise videos	3 (8.6)	4 (11.4)	13 (37.1)	13 (37.1)	2 (5.7)
I would also be willing to perform VR-supported physiotherapeutic exercises at home	—	2 (5.7)	6 (17.1)	20 (57.1)	7 (20)

a Six statements assessed motivation, perceived distraction from hospital routines, suitability of VR as a supplement or replacement for conventional physiotherapy, preference compared with traditional exercise media, and willingness to use VR at home. Values are presented as absolute frequencies (n) and percentages (%) based on a 5-point Likert scale.

b Not applicable.

**Figure 6.**
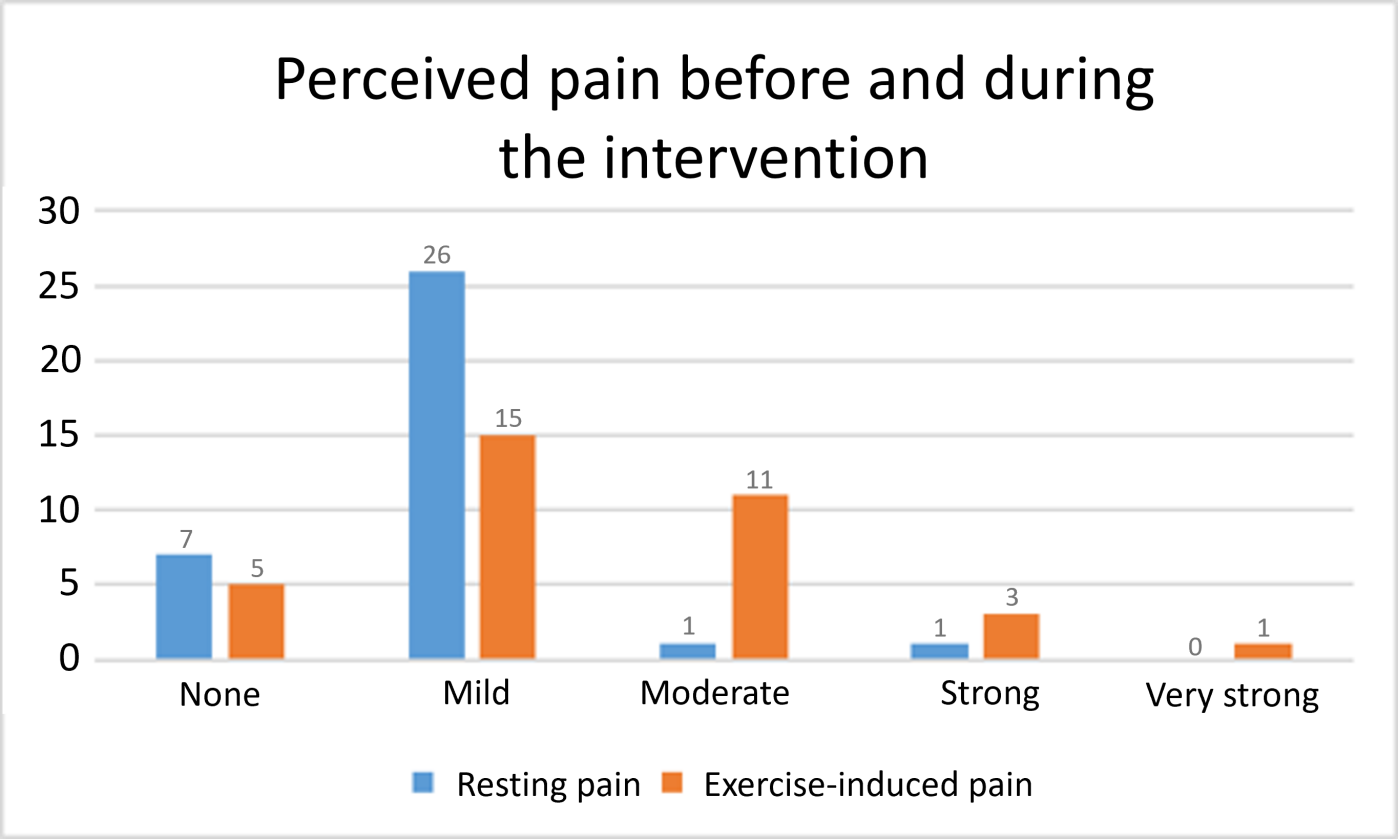
Patient-reported pain intensity levels before and during the virtual reality–supported physiotherapeutic exercises. The bar chart shows the distribution of self-reported pain intensity levels among postoperative patients (N=35), assessed at rest before the exercise session and during the exercise session. Pain intensity was categorized as none, mild, moderate, strong, or very strong.

Figure 6 illustrates the distribution of patients’ self-reported pain intensity levels before and during the VR-supported exercise session. Mean pain ratings increased from 0.89 (SD 0.58) before the exercise to 1.43 (SD 0.95) during the exercise. A Wilcoxon signed-rank test indicated a statistically significant within-subject difference between time points (*z*=3.49, *P*<.001, *r*=0.59), corresponding to a large effect size. Prior to the exercises, 26 out of 35 (74%) patients experienced mild pain, which decreased to 15 (43%) during the exercises.

One out of 35 (3%) patients reported moderate pain before the exercises, increasing to 11 (31%) during the exercises. Similarly, 1 (3%) patient reported strong pain before the exercises, which rose to 3 (9%) during the exercises. Only 1 (3%) individual reported very strong pain during the exercises. Additionally, 7 (20%) patients reported no pain before the exercises, while 5 (14%) reported no pain during the exercises. The responses from the 7 participating physiotherapists to a self-developed questionnaire are summarized in Table 3. The majority expressed a positive attitude toward VR-supported physiotherapy applications, with 6 participants indicating openness to such technologies and 5 envisioning their use in clinical practice. Regarding the potential of VR exercises to alleviate staffing shortages, most responses were neutral, while opinions on whether VR could reduce daily clinical workload varied between neutral and disagreement. Most physiotherapists agreed that, following adequate patient introduction, they could provide appropriate care using VR physiotherapy. Responses concerning VR’s ability to accommodate patient preferences were also predominantly neutral. Lastly, preferences between VR and more traditional alternatives, such as exercise videos, were mostly neutral among participants.

**Table 3.** Physiotherapists’ attitudes toward virtual reality (VR)–supported physiotherapeutic applications in a postoperative clinical pilot study^a^.

Question	Strongly disagree, n (%)	Disagree, n (%)	Neutral, n (%)	Agree, n (%)	Strongly agree, n (%)
I am open to a VR-supported physiotherapeutic application	—^b^	—	1 (14.3)	2 (28.6)	4 (58.1)
I can imagine working with VR in my practice	—	1 (14.3)	2 (28.6)	2 (28.6)	2 (28.6)
I believe that VR-supported exercises could help address understaffing within the team	1 (14.3)	1 (14.3)	4 (58.1)	—	1 (14.3)
I believe that VR-based physiotherapy could reduce workload in daily clinical routines	—	3 (42.9)	3 (42.9)	—	1 (14.3)
I believe that, following an appropriate patient introduction to VR physiotherapy, I could provide adequate patient care	—	—	5 (71.4)	1 (14.3)	1 (14.3)
I believe that VR physiotherapy could help accommodate patients’ preferences	—	—	4 (58.1)	2 (28.6)	1 (14.3)
I would prefer a more traditional alternative (eg, an exercise video) over VR	—	1 (14.3)	5 (71.4)	1 (14.3)	—

a Responses from physiotherapists (n=7) were collected as part of a prospective pilot study. A self-developed questionnaire was used to assess attitudes toward usability, feasibility of implementation, perceived workload implications, and preferences regarding VR-supported physiotherapy. Data are presented as absolute frequencies (n) and percentages (%) based on a 5-point Likert scale.

b Not applicable.

## Discussion

### Main Findings

This exploratory mixed methods study provides initial evidence that VR-based rehabilitation might generally be well accepted in an early postoperative clinical setting by both postoperative patients and physiotherapists. The average SUS scores for the overall system for both groups indicated an “above average” to “excellent” level of usability, according to SUS interpretation conventions. These categories are heuristic and should be interpreted cautiously, particularly in the absence of confidence intervals and given the pilot nature of the study.

In addition, no significant differences in perceived usability were found between age groups or with respect to gender. The general attitude toward VR usage in a clinical rehabilitation setting seems favorable for patients as well as for physiotherapists. As an exploratory pilot study, the present findings should be interpreted as hypothesis-generating and do not allow conclusions regarding clinical benefits or causal mechanisms.

Compared with the existing literature, this study provides initial results in a research field that has previously received little or no attention. Previous studies have primarily focused on other clinical domains, such as neurological or musculoskeletal rehabilitation, rather than early postoperative settings [17,18,28]. Therefore, even these results from this exploratory pilot study are highly interesting from a clinical perspective, since early postoperative mobilization is an important component of recovery, especially after abdominal or orthopedic procedures [29]. The integration of engaging, technology-supported interventions may enhance adherence and motivation [25].

Even participants 60 years and older provided favorable ratings, contrasting with previous reports indicating that the adoption of digital health technologies among older adults remains limited and selective [30].

Furthermore, the application was also perceived positively by physiotherapists, who play a central role in selecting and delivering therapeutic interventions. This observation is consistent with recent survey evidence indicating generally favorable clinician attitudes toward immersive VR, despite limited real-world adoption. For example, a cross-sectional international survey found that most physiotherapists perceived VR as clinically useful and not overly complex, and nearly all current users intended to continue using it, although concerns about cost and value for money were noted [31]. Similarly, a national survey in physiotherapy care reported that only a small minority of therapists currently use VR in practice, yet those who do report positive experiences and a willingness to recommend it, suggesting that adoption barriers may reflect structural rather than attitudinal limitations [25]. Their openness to VR in our study is encouraging, especially in light of the growing shortage of trained physiotherapy professionals in many health care systems. According to the World Health Organization and recent reports from national health agencies, the demand for rehabilitation services is increasing rapidly due to demographic aging, rising rates of chronic conditions, and a strained workforce [32-35]. Digital tools such as VR-based training might help in the future to bridge this gap by supporting therapists in repetitive or motivational aspects of care, thereby allowing them to allocate their time more effectively.

In addition to clinicians’ perspectives, patient-reported outcomes in this study further support the feasibility of VR-assisted rehabilitation. Most participants expressed favorable attitudes toward the system, reporting increased motivation, enjoyment, and willingness to use VR-based exercises as a supplement to conventional therapy. Notably, this positive appraisal persisted despite a statistically significant increase in pain ratings during task execution. On the one hand, this pattern might suggest that transient movement-related discomfort did not undermine acceptance. On the other hand, our data are consistent with rehabilitation contexts where temporary exertion-associated pain is expected during early mobilization via standard physiotherapy [36]. Rather than indicating a negative response to the intervention, the findings presented here may reflect typical activity-related sensations during therapeutic exercise, highlighting that experiential engagement and perceived usefulness can coexist with short-term physical strain.

### Limitations

Several methodological limitations should be noted. The small sample size and single-center design limit the generalizability of the findings. As SUS scores are influenced by system type and usage context, direct comparisons of absolute values across heterogeneous applications should be interpreted with caution [37].

Additionally, the study population may be subject to self-selection bias, as, first, individuals more comfortable with technology might have been more willing to participate, and second, only physically able patients were included. Both factors might potentially inflate usability ratings. The brief exposure time of 5 minutes to the VR application may not have allowed participants to fully familiarize themselves with the system, thus potentially impacting their subjective evaluations. Long-term acceptance and usability under routine clinical conditions remain to be explored.

Technical issues, including hardware limitations and software stability, were not extensively evaluated but may affect usability and integration into clinical workflows. Furthermore, adaptations might be necessary to accommodate diverse patient needs, particularly for older adults or those with physical impairments, to ensure equitable access and benefit.

### Implications for Future Research

Successful clinical implementation of VR-based rehabilitation requires appropriate training for both physiotherapists and patients to maximize engagement and therapeutic benefits [25]. The positive reception among physiotherapists suggests an openness to integrating VR tools into existing rehabilitation protocols, which may help address workforce shortages by supporting repetitive or motivational tasks.

In addition to the inherent limitations mentioned above, the observed usability and acceptance may partly be explained by XR-specific interaction characteristics such as spatial computing, immersive multisensory feedback, and embodied task execution, which can facilitate intuitive interaction, enhance engagement, and strengthen perceived task relevance. Prior research suggests that immersive environments and embodied interaction can increase motivation and user involvement by creating a stronger sense of presence and agency during task performance—factors that are considered central mechanisms underlying effective XR experiences [38]. However, as this study did not address important aspects that are known to play a key role in the acceptance of VR, namely spatial computing, immersion, or embodied interaction, and as these constructs were therefore not systematically measured or analyzed in relation to usability outcomes, this should be addressed in the future. Future studies should also incorporate validated XR-specific instruments to examine how such experiential factors may moderate acceptance and performance in clinical rehabilitation contexts.

Despite the drawbacks mentioned above, this study nevertheless provides some important insights. First, while most of the existing literature focuses on clinical effectiveness, this study adds to the limited body of research addressing technology acceptance and usability in surgical rehabilitation settings. Second, by incorporating both patients and clinical staff in the evaluation process, the study highlights the dual necessity of acceptance on both ends for successful clinical implementation and the resulting fact that VR must be carefully designed to complement rather than disrupt standard care pathways. Future studies should therefore investigate the long-term efficacy and user acceptance of VR rehabilitation over extended periods among larger, more diverse populations. Randomized controlled trials comparing VR-based interventions with traditional and alternative digital therapies are warranted to establish clinical effectiveness and best practice guidelines.

### Conclusions

This exploratory mixed methods study seems to indicate that VR-based rehabilitation could be generally well accepted by both postoperative patients and physiotherapists in the future, with usability ratings indicating an excellent level of user-friendliness across age groups and genders. The findings seem to suggest that VR applications can potentially serve as a feasible and engaging supplement to conventional physiotherapy in clinical settings. Notably, this study addresses a clinical context that has been underrepresented in prior VR rehabilitation research, namely early postoperative inpatient care. However, the limited sample size, brief exposure time, and single-center design indicate the need for further research.

Overall, the results of this pilot study warrant further investigation of VR applications, such as the one tested “Beer Pong” in larger clinical trials, with a focus on enhancing patient motivation and adherence, supporting physiotherapists, and potentially improving postoperative recovery outcomes. Larger randomized controlled trials with extended intervention periods are necessary to confirm these findings and evaluate long-term usability, clinical benefits, and cost-effectiveness aspects. By providing initial feasibility evidence from both patient and clinician perspectives under real clinical conditions, this study contributes preliminary insight into the practical implementation potential of immersive rehabilitation tools. In addition, larger trials should look into VR’s potential to support clinical workflows, especially amid workforce shortages.

## Supplementary material

10.2196/82704 Multimedia Appendix 1 Questionnaires.

## Abbreviations

ICH-GCP: International Council for Harmonization Good Clinical Practice
SUS: System Usability Scale
VR: virtual reality
