# Supplementary material for: User Acceptance of a Virtual Reality Beer Pong Intervention in Postoperative Rehabilitation: Exploratory Mixed Methods Study
Source: JMIR XR Spat Comput. 2026 Aug 6;3:e82704. doi: 10.2196/82704 (PMC13446801; doi:10.2196/82704)
Supplement: Supplementary file 1 [file xr-v3-e82704-s001.pdf]

Fragebogen Patient*innen

Alter:

Geschlecht:

Sportliche Tätigkeiten vor dem Krankenhausaufenthalt:

Erkrankung:

Therapie/operativer Eingriff:

Schmerzen in Ruhe (NSS):

Schmerzen bei Belastung bzw. während der Anwendung (NSS):

Haben Sie bereits Erfahrung mit Virtual Reality (VR): ja / nein

Wenn ja, wie häufig: <5 min 6-20 min >20 min

	Stimme überhaupt nicht zu	Stimme nicht zu	Teils / Teils	Stimme zu	Stimme voll und ganz zu
Verschiedene VR Szenarien zum Üben würden meine Motivation steigern	☐	☐	☐	☐	☐
Die Übungen mittels VR sind eine willkommene Ablenkung im Krankenhaus Alltag	☐	☐	☐	☐	☐
Ich kann mir vorstellen die Übungen mittels VR ergänzend zur üblichen Physiotherapie durchzuführen	☐	☐	☐	☐	☐
Ich kann mir vorstellen die Übungen mittels VR als Ersatz der üblichen Physiotherapie durchzuführen	☐	☐	☐	☐	☐
Ich bevorzuge VR zu herkömmlichen Alternativen z.B. das schauen und wiederholen eines Übungsvideos	☐	☐	☐	☐	☐
VR unterstützte physiotherapeutische Übungen würde ich auch zu Hause durchführen	☐	☐	☐	☐	☐

German System Usability Scale

Bitte überprüfen Sie die Antwort, die Ihre sofortige Antwort auf jede Aussage wiederspiegelt. Bitte denken Sie nicht zu lange über jede Aussage nach und stellen Sie sicher, dass Sie zu allen Aussagen eine Antwort gebe.

	Stimme überhaupt nicht zu	Stimme nicht zu	Teils / Teils	Stimme zu	Stimme voll und ganz zu
Ich denke, dass ich dieses Produkt häufig verwenden möchte	☐	☐	☐	☐	☐
Ich fand das Produkt unnötig komplex	☐	☐	☐	☐	☐
Ich dachte, das Produkt war einfach zu bedienen	☐	☐	☐	☐	☐
Ich denke, dass ich die Unterstützung einer technischen Person brauche, um dieses Produkt nutzen zu können	☐	☐	☐	☐	☐
Ich fand, die verschiedenen Funktionen in diesem Produkt waren gut integriert	☐	☐	☐	☐	☐
Ich dachte, dass dieses Produkt nicht konsistent genug war	☐	☐	☐	☐	☐
Ich würde mir vorstellen, dass die meisten Leute sehr schnell lernen würden, dieses Produkt zu benutzen	☐	☐	☐	☐	☐
Ich fand dieses Produkt sehr umständlich zu benutzen	☐	☐	☐	☐	☐
Ich habe mich sehr selbstsicher gefühlt, dieses Produkt zu benutzen	☐	☐	☐	☐	☐
Ich musste eine Menge Dinge lernen, bevor ich mit diesem Produkt loslegen konnte	☐	☐	☐	☐	☐

Haben Sie noch irgendwelche Anregungen oder Wünsche?

Weitere Kommentare?

Meiyuzi Gao , Philip Kortum & Frederick L. Oswald (2020): Multi-Language Toolkit for the System Usability Scale, International Journal of Human–Computer Interaction, DOI: 10.1080/10447318.2020.1801173

Fragebogen Physiotherapeuten

Alter:

Haben Sie bereits Erfahrung mit Virtual Reality (VR): ja / nein

Wenn ja, wie häufig: <5 min 6-20 min >20 min

	Stimme überhaupt nicht zu	Stimme nicht zu	Teils / Teils	Stimme zu	Stimme voll und ganz zu
Ich bin offen für eine VR unterstützte physiotherapeutische Anwendung	☐	☐	☐	☐	☐
Ich kann mir vorstellen mit VR zu arbeiten	☐	☐	☐	☐	☐
Ich kann mir vorstellen, dass VR gestützte Übungen einer Unterbesetzung im Team entgegen wirken könnten	☐	☐	☐	☐	☐
Ich kann mir vorstellen, das VR basierende Physiotherapie den Arbeitsallltag entlasten würde.	☐	☐	☐	☐	☐
Ich kann mich vorstellen, dass ich nach einer entsprechenden Einführung des Patienten mittels VR-Physiotherapie die Patienten gut betreuen kann	☐	☐	☐	☐	☐
Ich kann mir vorstellen, dass ich mittels VR-Physiotherapie den Patientenwünschen entgegenkommen kann	☐	☐	☐	☐	☐
Ich würde eine herkömmlichere Alternative (z.B. Übungsvideos) VR vorziehen	☐	☐	☐	☐	☐

Wie könnte eine Integrierung von VR gestützter Physiotherapie im Alltag ihrer Meinung nach aussehen?

German System Usability Scale

Bitte überprüfen Sie die Antwort, die Ihre sofortige Antwort auf jede Aussage wiederspiegelt. Bitte denken Sie nicht zu lange über jede Aussage nach und stellen Sie sicher, dass Sie zu allen Aussagen eine Antwort gebe.

	Stimme überhaupt nicht zu	Stimme nicht zu	Teils / Teils	Stimme zu	Stimme voll und ganz zu
Ich denke, dass ich dieses Produkt häufig verwenden möchte	☐	☐	☐	☐	☐
Ich fand das Produkt unnötig komplex	☐	☐	☐	☐	☐
Ich dachte, das Produkt war einfach zu bedienen	☐	☐	☐	☐	☐
Ich denke, dass ich die Unterstützung einer technischen Person brauche, um dieses Produkt nutzen zu können	☐	☐	☐	☐	☐
Ich fand, die verschiedenen Funktionen in diesem Produkt waren gut integriert	☐	☐	☐	☐	☐
Ich dachte, dass dieses Produkt nicht konsistent genug war	☐	☐	☐	☐	☐
Ich würde mir vorstellen, dass die meisten Leute sehr schnell lernen würden, dieses Produkt zu benutzen	☐	☐	☐	☐	☐
Ich fand dieses Produkt sehr umständlich zu benutzen	☐	☐	☐	☐	☐
Ich habe mich sehr selbstsicher gefühlt, dieses Produkt zu benutzen	☐	☐	☐	☐	☐
Ich musste eine Menge Dinge lernen, bevor ich mit diesem Produkt loslegen konnte	☐	☐	☐	☐	☐

Haben Sie noch irgendwelche Anregungen oder Wünsche?

Weitere Kommentare?

Meiyuzi Gao , Philip Kortum & Frederick L. Oswald (2020): Multi-Language Toolkit for the System Usability Scale, International Journal of Human–Computer Interaction, DOI: 10.1080/10447318.2020.1801173
